# A Step Toward a Global Consensus on Gastric Cancer Resectability Integrating Artificial Intelligence-Based Consensus Modelling

**DOI:** 10.3390/cancers17162664

**Published:** 2025-08-15

**Authors:** Katarzyna Gęca, Franco Roviello, Magdalena Skórzewska, Radosław Mlak, Wojciech P. Polkowski, ICRGC Collaborators

**Affiliations:** 1 Department of Surgical Oncology, Medical University of Lublin, 20-080 Lublin, Poland; magdalena.skorzewska@umlub.pl (M.S.); wojciech.polkowski@uml.edu.pl (W.P.P.); 2 General and Surgical Oncology Department, University of Siena, 53100 Siena, Italy; franco.roviello@gmail.com; 3 Department of Laboratory Diagnostics, Medical University of Lublin, 20-093 Lublin, Poland; radoslaw.mlak@umlub.pl

**Keywords:** gastric cancer, resectability, ICRGC project, artificial intelligence

## Abstract

Background: Surgical resection remains central to the curative treatment of locally advanced gastric cancer (GC), yet global variability persists in defining resectability, particularly in complex scenarios such as multivisceral invasion, positive peritoneal cytology (CY1), or oligometastatic disease. The Intercontinental Criteria of Resectability for Gastric Cancer (ICRGC) project was developed to address this gap by combining expert surgical input with artificial intelligence (AI)-based reasoning. Methods: A two-stage prospective survey was conducted during the 2024 European Gastric Cancer Association (EGCA) meeting. Fifty-eight surgical oncologists completed a 36-item questionnaire on resectability, strategy, and quality metrics. Subsequently, they reviewed AI-generated responses based on current clinical guidelines and completed a second round. Concordance between human and AI responses was classified as full, partial, or discordant, and changes in surgeon opinions were statistically analyzed. Results: Substantial agreement was observed in evidence-based domains. Seventy-nine percent of surgeons agreed with AI on distinguishing technical from oncological resectability. In cT4b cases, 61% supported restricting multivisceral resection to high-volume centers. Similar alignment was found in CY1 (54%) and N3 nodal disease (63%). Partial concordance appeared in areas requiring individualized judgment, such as peritonectomy or bulky-N disease. After AI exposure, surgeon responses shifted toward guideline-consistent decisions, including increased support for cytoreductive surgery only when CC0/1 was achievable and stricter classification of R2 resections as unresectable. Following AI exposure, 27.1% of surgeons changed at least one answer in alignment with AI recommendations, with statistically significant shifts observed in items related to surgical margin definition (*p* = 0.015), anatomical resection criteria (*p* < 0.05), and hospital stay benchmarks (*p* = 0.031). Conclusions: The ICRGC study demonstrates that AI-driven consensus modeling can replicate expert reasoning in complex surgical oncology and serve as a catalyst for harmonizing global practice. These findings suggest that AI-supported consensus modeling may complement expert surgical reasoning and promote greater consistency in decision-making, particularly in controversial or ambiguous cases.

## Simple Summary

Resectability assessment in advanced gastric cancer remains one of the most debated areas in surgical oncology, particularly in cases involving multivisceral invasion, positive peritoneal cytology, or limited metastases. Despite its critical role in treatment planning, there is no international consensus on resectability criteria, leading to heterogeneity in practice. To address this, an expert-led initiative of the Intercontinental Criteria of Resectability for Gastric Cancer (ICRGC) conducted a two-stage consensus study integrating surgical expertise with structured input from artificial intelligence. Responses from gastrointestinal surgeons were compared with AI-generated recommendations rooted in current clinical guidelines. The findings reveal a high degree of concordance in evidence-based domains and suggest that AI-supported reasoning may help guide complex decision-making and promote standardization. This study represents an initial step toward developing a harmonized, multidisciplinary framework for defining gastric cancer resectability across diverse clinical settings.

## 1. Introduction

Gastric cancer (GC) remains a significant global health burden, ranking as the fifth most common malignancy and the fourth leading cause of cancer-related mortality worldwide [1,2,3]. In 2020 alone, over one million new cases and approximately 770,000 deaths were recorded globally [2]. Although the incidence of non-cardia GC has declined in many regions—attributed to reduced Helicobacter pylori prevalence, improved food preservation, and dietary changes—an increasing trend in adenocarcinomas of the gastric cardia and esophagogastric junction has been observed, particularly in high-income countries [4].

Despite advancements in systemic therapies—including perioperative chemotherapy, immunotherapy, and biomarker-driven targeted agents—the prognosis for patients with locally advanced, resectable GC remains suboptimal [5,6,7,8]. In western countries, five-year survival rates typically range from 20% to 40%, and only a minority of patients achieve long-term survival following surgery [9].

Surgical resection, particularly when achieving an R0 (microscopically margin-negative) status, continues to be the cornerstone of curative intent treatment [2,9,10]. However, outcomes are heavily influenced by tumor biology, anatomical extent, response to neoadjuvant therapy, and institutional expertise. Importantly, the decision to operate depends not only on technical feasibility (i.e., whether the tumor can be removed surgically) but also on oncological appropriateness—whether the intervention can reasonably result in curative clearance. This distinction between technical and oncological resectability is well recognized but not uniformly applied across institutions [11,12].

Currently, no universally accepted criteria exist for determining GC resectability, resulting in significant variability in surgical decision-making and outcomes worldwide. Furthermore, factors such as bulky nodal disease, peritoneal involvement, or limited metastases (e.g., to the ovaries or liver) present controversial scenarios that challenge conventional resectability definitions [13,14].

To address this gap, the Intercontinental Criteria of Resectability for Gastric Cancer (ICRGC) project was initiated as part of an expert-driven initiative supported by the European Chapter of the International Gastric Cancer Association (IGCA). This collaborative effort brings together surgical oncology specialists and artificial intelligence (AI) to develop harmonized criteria for resectability in GC. This manuscript presents the results of a two-stage expert survey conducted during the 2024 European Gastric Cancer Association (EGCA) meeting in Siena, evaluating the concordance and divergence between surgeons and AI in defining resectability across various clinical scenarios.

By exploring the interplay between human expertise and AI-supported frameworks, this study seeks to lay the groundwork for a standardized, globally applicable set of criteria for resectability in advanced GC. The findings pave the way for a consensus-driven framework, enhancing surgical decision-making and improving patient outcomes globally.

## 2. Materials and Methods

### 2.1. Study Design and Participants

The ICRGC project was designed to evaluate global expert consensus on the criteria used to define resectability in advanced GC. A prospective, two-stage cross-sectional survey was conducted during the European Gastric Cancer Association (EGCA) meeting held in Siena, Italy, on 4–5 October 2024 [15].

A structured questionnaire was distributed to attending surgical oncologists. Eligibility was limited to specialists actively performing or supervising gastrectomies. Respondents remained anonymous, and participation was voluntary. A total of 58 (1 round) and 48 (2 round) fully completed surveys were included in the final analysis.

### 2.2. Survey Instrument

The questionnaire comprised 36 items, organized into five domains (Appendix A):

1. Definitions of resectability—including differentiation between technical and oncological resectability.
2. Surgical strategy in borderline and advanced cases—covering lymphadenectomy extent, multivisceral resections, peritonectomy, and metastasectomy.
3. Impact of preoperative therapies—evaluating how neoadjuvant chemotherapy or immunotherapy influences surgical decision-making.
4. Surgical quality metrics—such as morbidity, mortality, hospital stay, and institutional volume.
5. Resectability thresholds—exploring clinical scenarios with variable or controversial interpretations.

Additionally, demographic and practice data were collected, including years of experience, surgical technique expertise (open, laparoscopic, robotic), institutional case volume, and personal gastrectomy caseload.

The same questionnaire was later presented to a large language model, ChatGPT-4o, trained by OpenAI.

In Question 20, the phrase “more conservative approach” referred to limiting the anatomical extent of surgery—such as avoiding multivisceral or extended lymphadenectomy—if the tumor responded favorably to neoadjuvant therapy. It did not imply omission of surgery or curative intent.

### 2.3. AI Model Preparation

ChatGPT-4o (OpenAI, 2024) is a multimodal large language model with natural language reasoning capabilities, trained on publicly available biomedical literature, clinical guidelines, and surgical oncology reference texts up to June 2024. Before being administered the ICRGC survey, the model was:

- Primed using zero-shot prompting: without access to previous surgeon responses.
- Conditioned via structured prompts to mimic clinical reasoning by gastrointestinal oncology experts.
- Constrained to evidence-based frameworks, including NCCN, ESMO, Japanese Gastric Cancer Association (JGCA), and recent meta-analyses on GC resectability and multimodal therapy.

No fine-tuning or custom training was performed beyond the general GPT-4o pretraining. Responses were generated in a standardized environment and independently reviewed for internal consistency and evidence alignment.

### 2.4. Two-Stage Comparative Design

Following the initial collection of responses from surgical oncologists (Round 1), the same questionnaire was completed by the AI model, ChatGPT-4o, under standardized prompting conditions. These AI-generated answers were subsequently disclosed to the same group of surgeons, who were then invited to re-complete the survey (Round 2) with full knowledge that the responses they were reviewing originated from an artificial intelligence system.

This deliberate transparency was intended to assess whether exposure to AI-supported reasoning could influence or unify expert surgical judgment and areas of disagreement could be narrowed through reflection on algorithmic logic and evidence synthesis.

The design enabled the evaluation of baseline inter-surgeon consensus (Round 1), level of concordance between surgeons and AI, and the extent to which AI-informed reflection could shift clinical opinions (Round 2).

### 2.5. Statistical Analysis

The analytical approach consisted of two primary components: a descriptive analysis of the participant cohort and a comparative assessment of response patterns across the study arms. Baseline demographic and clinical variables—including years of surgical experience, proficiency in laparoscopic and robotic gastrectomy, institutional and individual procedure volumes, and geographic origin—were summarized using frequencies and proportions.

Response patterns from the survey were analyzed along three distinct axes: the initial responses from surgeons (prior to AI exposure), the answers generated independently by the ChatGPT-4o model, and the revised responses from the same group of surgeons after reviewing the AI-generated input. This structure allowed for direct comparison between pre-AI and post-AI surgeon responses, as well as alignment with AI itself.

In the analysis of multiple-choice items, the most commonly selected human responses were compared with the AI’s recommendations. Concordance was categorized as full agreement (identical dominant choices), partial agreement (responses differing in wording but similar in clinical logic), or disagreement (responses diverging in principle, priority, or intent).

To further characterize the influence of AI on clinical reasoning, a subgroup analysis was performed. This focused on identifying items in which consensus increased following exposure to AI input, clinical scenarios that remained controversial despite AI support, and specific domains where surgeons modified their responses toward greater alignment with AI recommendations.

Finally, all questions were thematically classified into categories of “concordant,” “partially concordant,” or “discordant” based on the degree of overlap and conceptual alignment between surgeon and AI responses. Detailed mappings of these classifications are provided in Table 1, Table 2 and Table 3.

Concordance between AI and surgeon responses was classified as follows:

- Full concordance: Identical selection and reasoning between AI and majority surgeon response.
- Partial concordance: Responses differed in wording but reflected similar clinical logic or therapeutic intent.
- Discordance: Contradictory or opposing decisions.

All AI responses were categorical (i.e., single or multiple-choice), matching the survey format given to human participants.

This study was exploratory in nature and aimed to generate preliminary consensus data. As such, no formal sample size calculation was performed. The final cohort size (n = 58) reflected all responses obtained before the EGCA meeting and was deemed sufficient for descriptive and trend-level analysis.

## 3. Results

### 3.1. Respondent Characteristics

The survey included 58 surgical oncologists with varied experience in gastric cancer (GC) surgery. Notably, 23.2% had over 20 years of operative experience, while 35.7% had less than 5 years. Over half (53.7%) had more than 5 years of laparoscopic gastrectomy experience, whereas robotic surgery was less common—49.1% had no exposure, and only 12.3% had more than 5 years of experience.

Institutional surgical volume was mostly medium to high, with 70.7% of respondents working in centers performing over 30 gastrectomies annually. Individually, 44.8% performed fewer than 10 cases per year, while 24.2% operated at higher personal volumes (≥21 cases/year).

Regarding geographic representation, 91.4% of respondents were based in Europe, reflecting the regional concentration of the EGCA symposium where the survey was distributed. Participants from Asia and South America each represented 3.5% of the cohort, while 1.7% were based in North America. This profile indicates a primarily European dataset, with limited but relevant input from non-European specialists.

### 3.2. Concordance Between Surgeons and AI

The comparative analysis revealed substantial agreement between surgical experts and the AI model (ChatGPT-4o) across multiple domains of GC resectability, particularly in areas supported by established clinical guidelines or strong levels of evidence (presented in Table 2). These findings indicate that AI, when trained on validated data and presented within clinical context, can effectively emulate expert reasoning in complex surgical oncology decision-making.

One of the strongest alignments emerged in the conceptual distinction between technical and oncological resectability. Seventy-nine percent of surgeons agreed with the AI that these represent fundamentally different decision axes: the former refers to anatomical operability, while the latter emphasizes the achievement of R0 resection with curative intent. Furthermore, 64% of respondents identified curative gastrectomy as being applicable exclusively in the oncological context, and an equal proportion associated palliative gastrectomy with symptom relief performed only when safety allows. Twenty-four percent considered reduction surgery reasonable for selected oligometastatic disease, consistent with AI’s nuanced inclusion of intent-based indications.

In the setting of multivisceral resection for locally advanced (cT4b) tumors, 61% of surgeons concurred with the AI model that such complex procedures should be restricted to high-volume centers with robust outcome monitoring and proven safety records. The logic of balancing oncological benefit with operative risk was consistently emphasized by both parties.

Regarding the appropriateness of gastrectomy with metastasectomy in cM1 tumors, 47% of surgeons selected the AI-aligned view that surgery is justified only when R0 resection or complete cytoreduction (CCR 0–1) is realistically attainable, with 44% offering a more permissive—but still evidence-grounded—opinion. This consensus reinforces the conditional nature of surgery in oligometastatic disease and the shared emphasis on meaningful oncological impact.

In patients with positive peritoneal cytology (CY1), 54% of surgeons endorsed the same position as AI: gastrectomy with extensive peritoneal lavage should only be performed after conversion to negative cytology through neoadjuvant systemic and/or intraperitoneal therapy. This approach aligns with contemporary evidence framing CY1 as a biologic marker of systemic disease rather than purely local dissemination.

For N3 bulky lymph node metastases, 63% of respondents supported the AI recommendation that extended lymphadenectomy (D2plus or D3) is appropriate only after major tumor regression following induction therapy. Similarly, in borderline resectable scenarios, 53% of surgeons agreed with AI that the extent of lymphadenectomy may be adapted to include extra-regional nodal dissection if there is a favorable response to preoperative therapy—further illustrating the importance of treatment response in defining surgical strategy.

There was also strong concordance in identifying markers of unresectability. Fifty-three percent of surgeons, in agreement with AI, considered extensive tumor invasion into adjacent structures to be technically prohibitive. Seventy-five percent cited encasement of major vascular structures, 47% noted para-aortic lymph node involvement, 40% pointed to distant metastases or peritoneal implants, and 45% included positive cytology as criteria disqualifying curative resection. These collective perspectives reinforce the alignment between evidence-based AI logic and human expertise in risk stratification and oncologic decision thresholds.

In terms of defining what constitutes multivisceral resection, surgeon responses closely reflected the AI classification. Distal pancreatectomy was endorsed by 91%, colonic resection by 96%, liver segmentectomy or more by 86%, pancreatoduodenectomy by 86%, splenectomy by 79%, and salpingo-oophorectomy with or without hysterectomy by 71%. These selections underline a shared understanding of the procedural scope necessary in cT4b resections, dependent on direct invasion and technical feasibility.

Agreement extended to anatomical considerations in defining resection boundaries for locally advanced tumors. In proximal tumors, 67% supported total gastrectomy, 62% selected distal pancreatectomy, 71% approved splenectomy, and 58% endorsed resection of the left liver lobe—findings that matched AI’s anatomic resection logic. For tumors in the middle third of the stomach, 80% of surgeons supported total gastrectomy, 73% endorsed distal pancreas resection, 76% spleen resection, and 60% resection of the left liver lobe. These selections further demonstrated strong alignment in adapting surgical plans to the location and extent of tumor infiltration.

Finally, there was mutual agreement on which anatomical structures are not typically dissected during standard gastrectomy. Between 37% and 52% of surgeons—reflecting responses consistent with AI—indicated that structures such as the intrathoracic esophagus, diaphragmatic crura, liver, pancreas, colon, para-aortic lymph nodes, ovaries, and parietal peritoneum should not be routinely addressed unless specifically indicated. This restraint reflects adherence to oncological principles, reserving extended resection only for direct invasion or strategic necessity.

In summary, the high degree of concordance between surgeon responses and AI outputs across well-defined surgical domains highlights the reliability of AI as a consensus-supportive tool. In structured, evidence-driven scenarios, both human and algorithmic reasoning arrived at convergent decisions, providing a potential foundation for standardizing surgical recommendations in gastric cancer management.

### 3.3. Areas of Disagreement Between Surgeons and AI

Despite notable concordance in several evidence-based domains, the analysis also revealed distinct areas of disagreement between surgical experts and the AI model (presented in Table 3). These differences were particularly evident in clinical contexts requiring nuanced judgment, situational interpretation, or intraoperative flexibility.

A major area of divergence involved the adjustment of resectability criteria following neoadjuvant systemic therapy. Among surveyed surgeons, 36% supported modifying the initial resectability criteria based on observed clinical or radiological response—particularly when guided by intraoperative pathology consultation. In contrast, 31% of surgeons favored maintaining unchanged thresholds regardless of treatment response, to preserve consistency. The AI model, however, adhered strictly to pre-established oncologic criteria, emphasizing that resectability should be determined by the capacity to achieve R0 resection, and that deviation from this principal risk’s overtreatment or inappropriate surgery. This reflects a broader philosophical difference between evidence-fixed logic and individualized interpretation of dynamic clinical scenarios.

Another point of discrepancy concerned the timing of determining surgical intent. The AI model consistently recommended deferring the declaration of curative versus palliative intent until after resection, citing the importance of complete intraoperative information, such as frozen section results, cytology, and visual assessment. Conversely, 45% of surgeons reported making this decision earlier—often preoperatively—based on imaging, clinical context, and pre-treatment MDT assessments. This highlights the human tendency to anticipate intraoperative outcomes and integrate them into procedural planning, even in advance of confirmatory findings.

Together, these areas of disagreement underscore the inherent difference between standardized, guideline-driven AI logic and the more fluid, case-dependent reasoning applied by experienced clinicians. While AI offers consistency and adherence to best-practice frameworks, surgical decision-making continues to rely heavily on situational awareness, judgment, and institutional context.

### 3.4. Partial Concordance Between Surgeons and AI

In several clinical domains, the comparison between surgeon responses and AI recommendations revealed partial concordance—indicating areas where alignment was present but not dominant, or where nuanced clinical context influenced variability in human judgment (presented in Table 4). These cases are particularly insightful for identifying gray zones where AI guidance can serve as a scaffold, but surgical decision-making still relies on individualized considerations.

One such domain was the role of surgical margins in defining non-resectability. When asked whether inability to achieve clear resection margins qualifies as a criterion for unresectability, surgeon responses varied: 24% cited macroscopically positive margins (R2) as disqualifying, 32% required frozen section confirmation, while 18% accepted even R2 resection if symptomatic relief was possible. The AI strongly favored the position that only R2 status qualifies as non-resectable, aligning with guideline-driven oncologic thresholds, but acknowledged some flexibility may be warranted in palliative contexts. This divergence underscores the balance between strict definitions and pragmatic, patient-centered choices.

Regarding the influence of postoperative morbidity and mortality data on surgical decision-making, 65% of surgeons emphasized the importance of tracking complications, and 32% acknowledged that risk may be justified if curative intent is preserved. The AI agreed that the potential survival benefit of multivisceral resection can outweigh procedural risks under selected conditions, illustrating overlapping but context-dependent reasoning.

In questions concerning hospitalization metrics, only 35% of surgeons considered a short hospital stay (e.g., ≤7 days) as a meaningful marker of surgical quality, while 26% selected longer benchmarks, and 16% aligned with the AI’s position that duration should vary depending on the type of gastrectomy performed (total vs. distal vs. proximal). This illustrates differing interpretations of recovery benchmarks in surgical oncology.

Concerning multivisceral resection for cT4b tumors, 46% of surgeons agreed with the AI that such procedures should only be performed in settings with demonstrably low morbidity and strong multidisciplinary support. However, 37% supported broader indications, reflecting differing levels of risk tolerance across institutions and personal experience.

Partial concordance was also observed in the justification of peritonectomy for patients with overt peritoneal metastases (P1 disease). While 37% of surgeons supported this intervention only in the context of achievable complete cytoreduction (CC0/CC1), the AI insisted on this as a strict precondition. Here, the AI’s algorithmic threshold was more rigid, while surgeons acknowledged additional intraoperative nuances.

In the evaluation of bulky-N disease, surgeon opinions were highly distributed: 46% supported including nodal encasement of arteries as a resectability criterion, 30% selected imaging-based assessments, and 22% agreed with the AI that major vessel involvement typically signifies non-resectability. AI expressed that response 5 was optimal overall, but recognized responses 3 and 7 could be valid depending on the case, reflecting a flexible understanding informed by surgical variability.

Anatomical resection choices also revealed partial alignment. For tumors at the esophago-gastric junction (EGJ), surgeons supported resections of the lower esophagus (73%), spleen (55%), and proximal stomach segments (40–55%), partially aligning with AI’s resection strategy, which prioritized oncologic adequacy without unnecessary organ sacrifice. Similarly, in distal stomach tumors, surgeon selections included total gastrectomy in poorly cohesive carcinomas (65%), distal pancreas (42%), and colon (73%), overlapping with AI’s more systematic but case-specific rationale.

In staging approaches, 34% of surgeons endorsed comprehensive laparoscopic evaluation of surrounding structures to determine resectability, and 27% specifically emphasized its value in assessing technical feasibility—closely mirroring the AI’s recommendation for broad intraoperative assessment. Nonetheless, the remaining responses reflect variability in how staging laparoscopy is employed globally.

In the classification of technical resectability, 39% of surgeons agreed with the AI that thorough intraoperative evaluation is essential, while others placed greater reliance on preoperative imaging or exploratory findings alone. Similarly, when asked about the appropriate point to define surgical intent (curative vs. palliative), 46% of surgeons considered such classification based on nodal involvement, while 26% aligned with the AI’s assertion that if achieving R0 is highly unlikely due to disease burden, proceeding with surgery may be oncologically unjustified.

Collectively, these domains of partial concordance underscore how AI systems trained on literature and guidelines can provide structured recommendations, while expert surgeons integrate additional contextual and intraoperative cues into their decision-making. In areas where flexibility is warranted—such as palliative intent, anatomical complexity, and nuanced staging—the integration of AI into multidisciplinary settings could enhance consistency while preserving necessary human adaptability.

### 3.5. Shift in Surgeon Responses Between Round 1 and Round 2

The two-stage design of the ICRGC study allowed for direct assessment of how exposure to AI-generated recommendations influenced surgical decision-making. In Round 1, surgeons responded independently based on their personal experience and institutional standards. In Round 2, the same questions were repeated, but participants were made aware that the provided answers originated from an AI system (ChatGPT-4o), trained on current clinical guidelines and literature (Table 5).

Across multiple domains, a measurable shift toward more conservative, guideline-concordant decision-making was observed after AI exposure.

A comparative analysis between the first and second rounds of the survey—conducted before and after exposure to AI-generated responses—revealed nuanced but meaningful shifts in clinical attitudes among participating surgeons. Although the overall direction of changes did not always reach statistical significance, trends indicated a gradual alignment toward more conservative and guideline-driven decision-making, particularly in complex or borderline scenarios.

In questions related to the definition and implications of resectability, responses remained largely consistent (e.g., 79.7% in Round 1 vs. 79.2% in Round 2 affirmed the distinction between technical and oncological resectability, *p* = 1.000). However, notable directional shifts were observed in more ambiguous domains. For instance, the proportion of surgeons endorsing R2 margins as a criterion for non-resectability increased from 24.1% to 39.6% (*p* = 0.0967), suggesting growing caution regarding oncological radicality.

Similarly, for multivisceral resection, support for limiting such interventions to centers with proven low morbidity/mortality rose from 60.3% to 77.1% (*p* = 0.0941), reflecting closer alignment with AI’s conditional approach to high-risk procedures. In the context of P1 peritoneal metastases, the percentage of surgeons advocating for cytoreductive surgery only if CC0/CC1 could be achieved increased from 8.6% to 20.8% (*p* = 0.0949), paralleling AI’s strict criteria.

The impact of neoadjuvant therapy also became more apparent. Surgeons who shifted from routine to more conservative interpretations of resectability criteria following preoperative treatment grew from 8.9% to 25.0% (*p* = 0.0344), while support for cautious intraoperative re-assessment declined (35.7% to 16.7%, *p* = 0.0450). This reflects a trend toward adopting AI’s evidence-oriented stance on resectability thresholds post-induction therapy.

Another relevant shift was seen in staging preferences and operative planning. A significantly larger proportion of respondents in Round 2 emphasized anatomical inspection of the pancreas (50.9% vs. 76.6%, *p* = 0.0083), liver (52.7% vs. 74.5%, *p* = 0.0260), and parietal peritoneum (43.6% vs. 72.3%, *p* = 0.0049) during laparoscopic exploration—reinforcing AI’s recommendation for comprehensive intraoperative staging.

In terms of surgical intent, fewer surgeons were willing to declare a curative approach at the outset of surgery after AI exposure (45.5% in Round 1 vs. 28.3% in Round 2), whereas more deferred the decision until after assessing oncological feasibility intraoperatively or pathologically.

While many *p*-values hovered just above traditional thresholds for significance, the repeated directional convergence across multiple questions supports the notion that AI exposure subtly influenced clinical reasoning—promoting more uniform, evidence-aligned decision-making patterns among experts.

## 4. Discussion

Surgical resectability remains the cornerstone of curative intent in locally advanced GC. However, the lack of internationally standardized criteria to define resectability continues to create substantial heterogeneity in clinical decision-making. The ICRGC project was designed to explore this variability and assess whether AI, specifically a large language model (ChatGPT-4o), could serve as a unifying decision-support tool in complex oncological scenarios.

### 4.1. Standardization Through AI: A New Pathway to Consensus

Our findings demonstrated notable variation in surgical opinions across a range of GC scenarios, particularly those lacking high-level evidence—such as peritoneal metastases (P1), positive peritoneal cytology (CY1), or oligometastatic disease. The implementation of a two-round design allowed us to examine the direct influence of AI on expert decision-making. After reviewing AI-generated responses—clearly identified as such—surgeons showed a measurable shift toward more conservative and guideline-concordant recommendations. This suggests that AI, when trained on current evidence and guidelines, can function as a catalyst for harmonizing expert-level decisions, especially in situations characterized by ambiguity or institutional variability.

These results align with broader efforts to integrate AI into evidence-based medicine. For example, the FUTURE-AI initiative emphasizes fairness, universality, traceability, usability, robustness, and explainability as the foundational principles for trustworthy medical AI deployment [9]. Similarly, the American Society for Gastrointestinal Endoscopy (ASGE) has recognized the potential of AI to enhance consistency in procedural care, stressing the need for consensus-driven quality benchmarks in endoscopic workflows [10].

### 4.2. Concordance and Divergence: Evidence-Based vs. Contextual Judgment

Surgeons and AI exhibited strong agreement in domains underpinned by robust evidence—such as the critical importance of achieving R0 resection, the role of diagnostic laparoscopy in staging, and the conceptual distinction between technical and oncological resectability. These findings support the notion that guideline-derived logic can be reliably reproduced by AI and accepted by experts in practice.

However, key areas of divergence remained, especially in borderline or high-risk cases. For instance, while AI applied strict criteria in scenarios involving CY1 or peritoneal disease—requiring cytologic conversion or complete cytoreduction (CC0/1)—many surgeons accepted broader indications based on intraoperative findings, institutional capacity, or palliative needs. Similarly, surgeons were more likely to declare curative versus palliative intent preoperatively, whereas AI deferred this decision until after resection, contingent on intraoperative confirmation.

These discrepancies highlight a fundamental tension: AI offers algorithmic consistency, but clinical decision-making still relies heavily on human adaptability, intraoperative assessment, and patient-centered reasoning. As Loftus et al. note, while AI can support efficiency and pattern recognition, its application in surgery must account for the complexity, uncertainty, and real-time variables inherent to the operating room [11].

The observed increase in selection of “more conservative approach” following AI exposure in Question 20 likely reflects support for tailoring surgical extent based on tumor response, not withholding surgery altogether. The AI interpretation remained consistent with oncologic principles of conversion surgery, supporting less invasive but still curative approaches in downstaged tumors.

While the present study demonstrates that AI exposure influenced surgeons toward more guideline-concordant decisions, the clinical implications of such shifts remain speculative. For example, refraining from surgery in cases of persistent CY1 status or unresectable nodal disease is consistent with evidence-based practice and may reduce non-beneficial surgery and associated morbidity.

However, strict adherence to AI-derived recommendations may carry a risk of over-conservatism, particularly in borderline resectable or oligometastatic scenarios where nuanced human judgment may allow for potentially curative interventions. AI’s current inability to integrate soft clinical variables (e.g., patient motivation, frailty, institutional expertise) underscores this risk.

Future prospective studies should evaluate whether AI-supported decision frameworks lead to improved clinical outcomes, such as higher R0 resection rates, reduced complications, or better survival. Only such outcome-linked validation can justify broader AI integration into oncologic decision-making.

### 4.3. Toward Hybrid Decision-Making Models

Our data reinforce the utility of a hybrid model—where AI serves as a reproducible, guideline-informed scaffold, while experienced clinicians retain flexibility to respond to patient-specific nuances. Such a model not only supports educational efforts for junior surgeons and consistency in low-volume centers but also offers a foundation for dynamic, “living” consensus frameworks like ICRGC.

This approach is also aligned with broader regulatory recommendations from the World Health Organization (WHO) and the International Telecommunication Union (ITU), which advocate for transparent, locally adaptable AI systems co-developed with clinicians to support equitable care across global healthcare systems [12].

### 4.4. Strengths and Limitations

This study possesses several notable strengths that reinforce the validity and relevance of its findings. Foremost, the prospective two-round design allowed for a unique within-subject comparison, directly measuring the influence of AI-generated input on expert surgical decision-making. By evaluating each surgeon’s responses before and after AI exposure, the study offers rare insight into how algorithmic recommendations shape clinical reasoning.

Moreover, the cohort was composed of experienced surgical oncologists from across the world, many of whom practice in high-volume centers and regularly manage complex GC cases. This lends the dataset high external validity within the context of advanced GC surgery. The methodological transparency—particularly the head-to-head comparison between surgeon-only and AI-assisted answers—also adds rigor and reproducibility, aligning the study with contemporary principles for AI evaluation in healthcare, such as those defined by the FUTURE-AI consortium.

However, several limitations should be acknowledged. The geographic composition of the cohort was predominantly European (91.2%), which may limit the global generalizability of the findings, particularly to settings with differing surgical infrastructure, case volumes, or training paradigms. Additionally, the AI model used in this study (ChatGPT-4o) has several important limitations. It operates as a generalist language model with a knowledge cutoff of June 2024 and lacks access to real-time clinical data, institutional protocols, or intraoperative findings [16,17]. As such, its responses are grounded in synthesized guideline-based evidence but may not reflect emerging practice patterns or individualized patient considerations. Furthermore, ChatGPT-4o is not fine-tuned for surgical oncology decision-making and cannot dynamically adapt to new data [18]. Future work may explore the role of adaptive AI frameworks capable of integrating structured clinical input, outcome feedback, and continuous learning to enhance clinical relevance [19,20].

Furthermore, the study was based on a survey format, and while scenarios were clinically realistic, they did not fully replicate the complexity and time-sensitive pressures of live intraoperative decision-making. Finally, the absence of randomization or blinding in the survey design introduces the possibility of anchoring bias or cognitive priming, particularly in the second round where participants were aware that responses were AI-generated.

Although subgroup comparisons by surgeon experience or annual case volume could provide valuable insights, the sample size in this study was insufficient to support statistically meaningful stratified analyses. Future phases of the ICRGC project aim to include a larger and more diverse cohort, enabling more detailed exploration of how AI influences decision-making across different levels of surgical expertise.

As AI becomes more integrated into clinical environments, its role should remain complementary, particularly useful in low-volume centers or among junior clinicians, to support consistency and guideline adherence. However, reliance on AI must not stifle clinical innovation or replace the essential role of prospective clinical trials in advancing surgical oncology. Additionally, ethical and implementation challenges, including medicolegal responsibility, data privacy, and workflow integration, must be addressed before routine clinical deployment. These issues warrant further investigation and careful design of future AI-enabled clinical tools. These issues have been widely discussed in the literature, particularly regarding the balance between AI-assisted consistency and clinical autonomy [17,21,22], and the importance of regulatory, ethical, and legal frameworks for implementation [23,24].

The absence of a formal power calculation represents a limitation of this exploratory study. While trends and concordance levels were observed, the sample size may limit the ability to detect small but potentially meaningful differences in subgroup comparisons or response shifts.

The absence of tumor biology variables in the resectability scenarios is one of this study’s limitations. While anatomical extent and imaging-based criteria remain critical for surgical planning, emerging evidence supports the prognostic and predictive relevance of molecular subtypes—such as HER2 amplification, microsatellite instability (MSI), and histologic type (e.g., diffuse vs. intestinal Lauren classification) [10,25,26,27]. These factors increasingly influence neoadjuvant therapy choices and surgical decisions. Future iterations of the ICRGC survey should integrate such variables to reflect the evolving biology-driven landscape of gastric cancer care.

Another limitation is the potential influence of disclosing the AI origin of Round 2 responses. Although surgeons were aware that the recommendations came from ChatGPT-4o, they were not informed about how the AI generated its outputs [28]. This disclosure may have introduced an authority bias, whereby participants adjusted their answers due to the perceived credibility of AI rather than the content itself [29,30,31,32]. This effect was not formally assessed and represents an important consideration for future research designs aiming to evaluate AI’s influence on clinical judgment.

Despite these limitations, the study represents an important first step in evaluating how structured AI systems can influence expert-level surgical consensus, and it provides a valuable foundation for future investigations in real-world, multidisciplinary contexts.

## 5. Conclusions

This study demonstrates that AI-guided consensus modeling can support—but not replace—expert clinical reasoning in complex surgical oncology scenarios. AI-based recommendations showed high concordance with human responses in guideline-driven areas and modestly influenced decision-making in ambiguous cases. These findings suggest that AI frameworks may serve as complementary tools within multidisciplinary teams, facilitating standardization and documentation of clinical reasoning. Future integration should proceed cautiously, addressing legal, ethical, and workflow considerations, and ideally begin within structured environments such as tumor boards.

## Figures and Tables

**Table 1:** Characteristic of respondent group.

Answer	Before AI (First Round)	
	N	(%)
Question 26: Please describe your experience with performing gastrectomy as an 1st operator: (n = 56)		
Less than 5 years	20	35.71%
5–10 years	6	10.71%
10–15 years	9	16.07%
15–20 years	8	14.29%
More than 20 years	13	23.21%
Question 27: What is your experience in laparoscopic gastrectomy: (n = 57)		
No experience	8	14.04%
Less than 5 years	16	28.07%
5–10 years	18	31.58%
10–15 years	9	15.79%
15–20 years	3	5.26%
More than 20 years	3	5.26%
Question 28: What is your experience in robotic gastrectomy: (n = 57)		
No experience	28	49.12%
Less than 1 year	12	21.05%
1–5 years	8	14.04%
5–10 years	7	12.28%
10–20 years	2	3.51%
More than 20 years	0	0.00%
Question 29: Do you perform staging laparoscopy in patients with gastric cancer? (n = 57)		
Yes, routinely in patients with “non-early” GC who are not amenable to endoscopic treatment and who do not have clinically (imaging) detectable distant metastases (cM0)	31	54.39%
Yes, but only when non-resectability is suspected	10	17.54%
Yes, I start every gastrectomy for advanced GC with laparoscopy	5	8.77%
Yes, twice: as a staging procedure (before creation of treatment plan by the MDT), and after preoperative (neo-adjuvant) therapy–re-staging	11	19.30%
No, never	0	0.00%
Question 30: Do you perform extended D2plus/D3 lymphadenectomy with removal of extra-regional lymph nodes (i.e., para-aortic etc.)? (multiple choice) (n = 57)		
Yes	7	12.28%
Sometimes	13	22.81%
If a positive para-aortic node is found (longest axis at least 10 mm) at preoperative radiological examination	5	8.77%
In patients who have radiological evidence of positive para-aortic nodes before preoperative chemotherapy and then show clinical response at restaging examinations	24	42.11%
Only when lymph node metastases are proved (intraoperative pathology counselling)	1	1.75%
Even if these lymph nodes are not suspected for metastases	1	1.75%
Even if these lymph nodes are not suspected for metastases, but the patient shows clinical response to preoperative chemotherapy	1	1.75%
No, never	5	8.77%
Question 31: Do you perform multivisceral resection for cT4B gastric cancer? (n = 57)		
Yes	9	15.79%
Sometimes	20	35.09%
Only when direct invasion of adjacent organ is proved (intraoperative pathology counselling)	16	28.07%
Even when direct invasion of adjacent organ is not suspected (preoperative imaging), but I have such a suspicion during gastrectomy	10	17.54%
No, never. It is not justified	2	3.51%
Question 32: Do you extend gastrectomy with metastasectomy (liver, adrenalectomy, ovaries, other organs) for M1 gastric cancer? (n = 56)		
Yes	6	10.71%
Sometimes	17	30.36%
Only when M1 is proved by intraoperative pathology	1	1.79%
Only if it fulfils criteria of oligometastatic disease (OMEC definition)	31	55.36%
No, never. It is not justified	1	1.79%
Question 33: Do you extend gastrectomy with peritonectomy for P1 gastric cancer (with overt peritoneal metastasis)? (n = 57)		
Yes, but only within a clinical trial	10	17.54%
Only when PCI is no more than 6	14	24.56%
Only when PCI is no more than 12 for non-poorly cohesive tumours	1	1.75%
Only if the procedure can be supplemented with the HIPEC	21	36.84%
No, never. It is not justified	11	19.30%
Question 34: Do you perform gastrectomy for positive cytology (CY1) gastric cancer without peritoneal metastases at preoperative staging? (n = 58)		
Yes always, but only within a clinical trial	6	10.34%
Only when positive cytology is reversed to negative by preoperative (induction) systemic therapy	24	41.38%
Even when peritoneal cytology still positive after preoperative systemic therapy	7	12.07%
Only if the procedure can be supplemented with the HIPEC	20	34.48%
No, never. It is not justified	1	1.72%
Question 35: What is your institution yearly gastrectomy volume? (n = 58)		
<20	5	8.62%
20–30	12	20.69%
31–50	19	32.76%
51–100	14	24.14%
>100	8	13.79%
Question 36: What is your personal yearly gastrectomy volume? (n = 58)		
<10	26	44.83%
10–20	13	22.41%
21–30	5	8.62%
31–50	11	18.97%
>50	3	5.17%
Question 37: What continent do you come from? (n = 58)		
Asia	2	3.45%
North America	1	1.72%
South America	2	3.45%
Europe	53	91.38%
other	0	0.00%

**Table 2:** Concordance between surgeons and AI answers.

No	Question	Surgeons	AI
1	Resectability is to be decided based on preoperative imaging, staging laparoscopy findings and intraoperative findings (clinical stages, cTNM, cStage). Do you think terms technical resectability & oncological resectability are different:(multiple choices are relevant)	1—Yes, they are—79% 3—Curative gastrectomy refers only to oncological resectability—64% 4—Palliative gastrectomy is for symptom relief only when safe – 64% 6—Reduction gastrectomy after response to preoperative chemotherapy—24%	1—Yes, they are (technical resectability & oncological resectability are different) 3—Curative gastrectomy (performed with the intention of cure) refers only to oncological resectability 4—Palliative gastrectomy (when cure is not possible) is performed to relieve patients (serious) symptoms only when it may be done safely 6—Reduction gastrectomy should be performed for oligometastatic disease with partial response to preoperative chemotherapy
4	Tracking the rate of morbidity/mortality following gastrectomy is an important measure of surgical quality and patient safety. Whether these outcome measures should influence a decision on performance of gastrectomy with multivisceral resection?	3—Multivisceral resections for locally advanced gastric cancer should only be undertaken in centres that carefully monitor their own results and can demonstrate acceptably low morbidity/mortality associated with gastrectomy and multivisceral resection—61%	3—Multivisceral resections for locally advanced gastric cancer should only be undertaken in centres that carefully monitor their own results and can demonstrate acceptably low morbidity/mortality associated with gastrectomy and multivisceral resection
7	Is gastrectomy with metastasectomy justified for cM1 tumours	3—Only if R0 or complete cytoreduction group (CCR 0–1) is anticipated to achieve—47% 4—Only if the stage does not exceed (generally accepted) definition of the oligometastatic disease—44%	3—Only if R0 or complete cytoreduction group (CCR 0–1) is anticipated to achieve Reason: R0 resection or complete cytoreduction (CCR 0–1) is essential for considering gastrectomy with metastasectomy in cM1 tumors. Achieving complete removal of all visible disease significantly improves prognosis in selected cases. This aligns with the principle that surgery is justified only if it contributes meaningfully to disease control.
9	Is gastrectomy with extensive peritoneal lavage justified for CY1 tumours (with positive peritoneal cytology):	5—Only if the resection is preceded by conversion to negative peritoneal cytology under the induction (neoadjuvant) systemic [with/without intraperitoneal therapy (i.p. normothermic paclitaxel/PIPAC)] therapy –54%	5—Only if the resection is preceded by conversion to negative peritoneal cytology under the induction (neoadjuvant) systemic [with/without intraperitoneal therapy (i.p. normothermic paclitaxel/PIPAC)] therapy Reason: Positive peritoneal cytology (CY1) is a marker of poor prognosis. Conversion to negative cytology after neoadjuvant systemic or intraperitoneal therapy significantly improves the likelihood of achieving long-term survival. Gastrectomy with peritoneal lavage is justified in this context as part of a comprehensive treatment strategy aimed at achieving R0 resection.
10	Is gastrectomy with extended (D2plus/D3) lymphadenectomy justified for N3 bulky lymph node metastasis:	2—Only if the resection is preceded by major regression under the induction (neoadjuvant) systemic (immuno-)therapy—63%	2—Only if the resection is preceded by major regression under the induction (neoadjuvant) systemic (immuno-)therapy Reason: N3 bulky lymph node metastasis represents advanced nodal disease and is often associated with poor prognosis. Significant regression after neoadjuvant systemic or immunotherapy improves the feasibility of achieving an R0 resection and may justify an extended lymphadenectomy. Without such regression, surgery is unlikely to provide meaningful survival benefits and carries higher risks of morbidity.
11	Evaluating the extent and thoroughness of lymph node dissection is important for staging and determining the prognosis of the patient. If a case is borderline resectable at your initial (after exploration) evaluation, should it modify the extent of ultimate lymphadenectomy:	6—Yes, it should be D2 plus (extraregional lymph nodes) if there is a good response to preoperative therapy—53%	6—Yes, it should be D2 plus (extraregional lymph nodes) if there is a good response to preoperative therapy Reason: In borderline resectable cases, the response to preoperative therapy is a critical determinant for the extent of lymphadenectomy. If there is a favorable response (e.g., significant tumor regression), an extended lymphadenectomy (D2 plus) may improve staging accuracy and potentially enhance survival outcomes. However, the decision must be weighed against the patient’s condition and perioperative risks.
13	Would you call it multivisceral resection? Gastrectomy with:(multiple choices are relevant)	3—Right and/or left diaphragmatic crus—25% 4—Splenectomy– 79% 5—Distal pancreatectomy– 91% 6—Liver resection with one/two liver segments—86% 7—Liver resection with three or more liver segments—63% 8—Pancreatoduodenectomy—86% 9—Colonic resection (hemicolectomy or transversectomy)—96% 10—Salpingo-oophorectomy with or without hysterectomy—71%	3—Right and/or left diaphragmatic crus 4—Splenectomy 5—Distal pancreatectomy 6—Liver resection with one/two liver segments 7—Liver resection with three or more liver segments 8—Pancreatoduodenectomy 9—Colonic resection (hemicolectomy or transversectomy) 10—Salpingo-oophorectomy with or without hysterectomy
15	Please indicate structures (that may be directly invaded; cT4b) to be resected if primary tumour is located at the proximal stomach (not invading esophago-gastric junction): (multiple choices are relevant)	1—Lower esophagus (transhiatally)—58% 2—Whole stomach—67% 3—Distal pancreas—62% 4—Spleen—71% 6—Proximal two/thirds of the stomach—24% 7—Left liver lobe—58%	2—Whole stomach Reason: In some cases, resection of the entire stomach may be necessary for oncological clearance, especially if the tumor extensively involves the proximal stomach and other regions. 3—Distal pancreas Reason: Tumors located in the proximal stomach may invade the distal pancreas, particularly if they are in the posterior wall or near the greater curvature. 4—Spleen Reason: Due to its proximity to the proximal stomach, the spleen may be invaded by tumors extending laterally or posteriorly. 6—Proximal two/thirds of the stomach Reason: This is a common extent of resection for tumors confined to the proximal stomach that do not require total gastrectomy. 7—Left liver lobe Reason: Tumors in the proximal stomach, particularly those near the lesser curvature, may invade the left lobe of the liver.
16	Please indicate structures (that may be directly invaded; cT4b) to be resected if primary tumour is located at the middle third of the stomach: (multiple choices are relevant):	2—Whole stomach—80% 3—Distal pancreas—73% 4—Spleen—76% 6—Left liver lobe—60%	2—Whole stomach Reason: Tumors in the middle third of the stomach often require total gastrectomy for adequate oncological clearance if they extend significantly proximally or distally. 3—Distal pancreas Reason: The tumor’s location near the posterior wall or greater curvature can lead to invasion of the distal pancreas, necessitating resection. 4—Spleen Reason: Tumors involving the posterior or lateral portions of the middle third of the stomach may invade the spleen, especially near the greater curvature. 5—Proximal one/third of the stomach Reason: Tumors extending proximally may require resection of the proximal stomach to achieve clear margins. 6—Left liver lobe Reason: Tumors on the lesser curvature of the stomach can invade the left liver lobe, particularly with posterior extension.
19	Gastric adenocarcinomas are considered unresectable if:	1—The primary tumor shows extensive invasion to adjacent structures and cannot be separated from the surrounding normal tissues—53% 2—The primary tumor has encased major vascular structures—75% 4—Presence of metastatic lymph nodes outside the scope of surgery—47% 5—Presence of distant metastasis or intraperitoneal implantation (including positive peritoneal lavage fluid cytology—40% 6—presence of distant metastasis or intraperitoneal implantation (excluding positive peritoneal lavage fluid cytology) –45%	1—The primary tumor shows extensive invasion to adjacent structures and cannot be separated from the surrounding normal tissues Reason: Extensive invasion into adjacent structures that cannot be separated indicates technical non-resectability due to the impossibility of achieving R0 resection without compromising critical structures. 2—The primary tumor has encased major vascular structures Reason: Encasing major vascular structures (e.g., aorta, celiac axis) makes the tumor technically non-resectable and increases the risk of incomplete resection (R2) or patient morbidity/mortality. 4—Presence of metastatic lymph nodes outside the scope of surgery Reason: Metastatic lymph nodes outside the planned surgical field (e.g., para-aortic nodes) suggest systemic disease, making the tumor oncologically unresectable. 5—Presence of distant metastasis or intraperitoneal implantation (including positive peritoneal lavage fluid cytology) Reason: Distant metastases or peritoneal involvement indicate systemic disease, which precludes curative resection.
23	Structures for which otherwise no further dissection is necessary during standard gastrectomy include:	1—Intrathoracic esophagus—39% 2—Diaphragmatic crura—37% 3—Liver—52% 4—Spleen– 50% 5—Pancreas—50% 6—Colon—48% 7—Para-aortic lymph nodes– 44% 8—Ovaries—46% 9—Greater omentum– 30% 10—Parietal peritoneum—43%	1—Intrathoracic esophagus Reason: Dissection of the intrathoracic esophagus is not part of a standard gastrectomy unless the tumor involves the esophago-gastric junction. 2—Diaphragmatic crura Reason: The diaphragmatic crura are not dissected during a standard gastrectomy unless there is evidence of tumor invasion or proximity to the esophago-gastric junction. 3—Liver Reason: The liver is not typically dissected during standard gastrectomy unless there is direct tumor invasion or metastatic disease requiring resection. 5—Pancreas Reason: The pancreas is not dissected in a standard gastrectomy unless there is tumor involvement or extension near the posterior gastric wall. 6—Colon Reason: The colon is not dissected unless there is direct tumor invasion requiring en bloc resection. 7—Para-aortic lymph nodes Reason: Dissection of para-aortic lymph nodes is not part of standard gastrectomy. It may be performed as part of an extended lymphadenectomy or if these nodes are involved. 8—Ovaries Reason: Ovaries are not routinely addressed during standard gastrectomy unless there are suspected or confirmed metastatic lesions (e.g., Krukenberg tumor). 10—Parietal peritoneum Reason: The parietal peritoneum is not typically dissected unless there is evidence of tumor invasion or peritoneal metastases.

**Table 3:** Areas of Disagreement Between Surgeons and AI.

No	Question	Surgeons	AI
20	Would you consider all the above resectability criteria the same if a neoadjuvant systemic (chemo-/immuno) therapy had been used before?	1—Yes, no matter preoperative treatment—31% 3—Yes, but with cautious use of intraoperative pathology counselling—36%	4—More conservative approach is justified if clinical response had been achieved Reason: Neoadjuvant systemic therapy can downstage tumors, reducing the extent of disease and potentially converting some cases previously deemed unresectable to resectable. A conservative surgical approach may be justified to balance the benefits of surgery against the risks, especially in patients demonstrating a good clinical response. This approach prioritizes achieving R0 resection without excessive morbidity.
25	At what point during the surgical process should the operating surgeon report whether the gastric cancer surgery is intended to be curative or palliative?	1—At the beginning of the procedure—45% 4—Only after evaluation of the final pathology report of the resection specimen, irrespectively of the peritoneal (lavage) cytology findings—25%	3—During the procedure, usually immediately following the resection Reason: The decision on whether the surgery is curative or palliative is most accurately determined during the procedure, particularly after the resection is performed. At this point, the surgeon has the most comprehensive intraoperative information, including the extent of tumor invasion, feasibility of achieving R0 margins, and findings from staging evaluations such as frozen section results or peritoneal lavage cytology. This allows for a real-time, informed decision.

**Table 4:** Partial Concordance Between Surgeons and AI answers.

No	Question	Surgeons	AI
2	Determining whether the surgical margins are clear of cancer cells by examining the resected tissue (frozen section) helps assess the oncological effectiveness of the procedure. Inability to achieve clear resection margins is a criterion of non-resectability.	1—Yes, but only macroscopically positive margins (R2)—24% 2—Yes, but only when you prove it with an intraoperative frozen section—32% 3—No, even when intraoperative frozen section is positive, because final pathology of the resection specimen may be different (clear margins)– 18% 5—Resections margins should be clear at every cost, and they are not a non-resectability criterion—18%	1—Yes, but only macroscopically positive margins (R2) 2—Yes, but only when you prove it with an intraoperative frozen section 4—No, even R2 resection is justified if it may eliminate symptoms
3	Achieving R0 resection status is critical for optimizing survival outcomes, despite the inherent risks and complications associated with extensive surgical interventions. Therefore, monitoring the occurrence of postoperative complications (morbidity) and mortality provides insight into the quality of perioperative care.	1—Risk of morbidity/mortality should always be in mind before decision of multivisceral resection—65% 3—Risk of morbidity/mortality associated with multivisceral resection may be outweighed by the benefits of improved survival after curative gastrectomy—32%	3—Risk of morbidity/mortality associated with multivisceral resection may be outweighed by the benefits of improved survival after curative gastrectomy
5	A shorter hospital stay is generally associated with better surgical outcomes and may indicate a more efficient recovery process. Therefore, duration of hospital stay for oncological gastrectomy should be below:	1—10 days—26% 6—Depends on total, distal or proximal gastrectomy—16% 7—Depends on splenectomy, pancreatectomy, any multivisceral resection, or HIPEC—35%	6—Depends on total, distal or proximal gastrectomy
6	Is gastrectomy with multivisceral resection justified for cT4b tumours (that invades adjacent structures such as spleen, transverse colon, liver, diaphragm, pancreas, abdominal wall, adrenal gland, kidney, small intestine, or retroperitoneum):	3—Only if R0 is anticipated to achieve—37% 5—Only if surgical team can prove low morbidity/mortality, as well as the multidisciplinary team can ensure that all aspects of the patient’s condition are considered—46%	5—Only if surgical team can prove low morbidity/mortality, as well as the multidisciplinary team can ensure that all aspects of the patient’s condition are considered
8	Is gastrectomy with peritonectomy justified for P1 tumours (with overt peritoneal metastasis):	1—Yes, but only for P1a (previous P1—i.e., disseminating metastasis to the region directly adjacent to the peritoneum of the stomach, including the greater omentum; not distant peritoneum or ovarian metastases)—16% 3—Yes, but only for PCI below 12, with poorly cohesive type below 7—16% 7—Only if the resection is preceded by major regression under the induction (neoadjuvant) systemic [with/without intraperitoneal therapy (i.p. normothermic paclitaxel/PIPAC)] therapy—37%	5—Only if complete cytoreduction (CC0/CC1) is to be achieved Reason: Achieving complete cytoreduction (CC0/CC1) is a prerequisite for considering gastrectomy with peritonectomy in patients with P1 tumors. Without complete cytoreduction, the procedure does not confer significant survival benefits and exposes patients to unnecessary surgical morbidity. AI considers response 5 to be the most appropriate overall, although responses 3 and 7 are also relevant in the context of specific clinical scenarios. If required to select a single option, AI would identify response 5 as the optimal choice.
12	Extensive nodal swelling along the major branched arteries or para-aortic lymph node swelling is called bulky-N disease. If large arteries are surrounded by bulky nodal metastatic tissue, do you consider the case resectable? Please indicate non-resectability (multiple choices are relevant):	1—Coeliac axis—46% 2—Common hepatic a.—30% 5—any of the above—22%	5—Any of the above Reason: If bulky-N disease involves extensive nodal swelling surrounding any of the major branched arteries (coeliac axis, common hepatic artery, left gastric artery, or proximal portion of the splenic artery), it typically renders the case non-resectable due to the high risk of incomplete resection (R2) and the technical challenges associated with safely dissecting these vessels. Such involvement also indicates an advanced stage of disease with poor prognosis, where surgery is unlikely to provide meaningful survival benefit.
14	Please indicate structures (that may be directly invaded; cT4b) to be resected if primary tumour is located at the esophago-gastric junction: (multiple choices are relevant):	1—Intrathoracic esophagus (below carina)—56% 2—Lower esophagus (transhiatally)– 73% 3—Whole stomach—46% 4—Distal pancreas—44% 5—Spleen—55% 6—Proximal one/third of the stomach—40% 7—Proximal two/thirds of the stomach—26% 8—Left liver lobe—55%	1—Intrathoracic esophagus (below carina): Reason: Tumors at the esophago-gastric junction (EGJ) often extend into the lower thoracic esophagus. If directly invaded, resection of this segment is necessary. 2—Lower esophagus (transhiatally): Reason: Transhiatal resection of the lower esophagus is a standard part of surgery for EGJ tumors with esophageal invasion. 3—Whole stomach: Reason: Depending on the extent of the tumor and gastric involvement, resection of the entire stomach may be required for oncological clearance. 5—Spleen: Reason: The spleen may be involved in cases of advanced tumors extending posteriorly or laterally. Resection may be necessary if invaded. 6—Proximal one/third of the stomach: Reason: If the tumor is localized to the EGJ and proximal stomach, resection of this portion is sufficient. 7—Proximal two/thirds of the stomach: Reason: In cases where the tumor involves a larger portion of the stomach but spares the distal part, this extent of resection may be warranted.
17	Please indicate structures (that may be directly invaded; cT4b) to be resected if primary tumour is located at the distal stomach:(multiple choices are relevant):	1—Whole stomach, if poorly cohesive carcinoma—65% 2—Distal pancreas—42% 3—Spleen– 36% 4—Distal three/thirds of the stomach (small remnant)—58% 5—Distal stomach—42% 6—Antrum of the stomach—31% 7—Colon—73% 8—Head of pancreas—53%	1—Whole stomach, if poorly cohesive carcinoma Reason: Poorly cohesive carcinomas (e.g., signet-ring cell carcinoma) often exhibit diffuse spread, making total gastrectomy necessary even for distal tumors to achieve R0 resection. 2—Distal pancreas Reason: Tumors in the distal stomach, particularly those involving the posterior wall or greater curvature, can invade the distal pancreas, necessitating resection. 4—Distal three/thirds of the stomach (small remnant) Reason: When oncological clearance requires resecting most of the stomach, leaving only a small remnant may be appropriate for proximal margin control. 5—Distal stomach Reason: This is the standard resection for tumors confined to the distal stomach that do not require total gastrectomy. 7—Colon Reason: Tumors in the distal stomach, particularly near the greater curvature or posterior wall, may invade the transverse colon, necessitating colonic resection.
21	Should staging laparoscopy be used for reliable resectability determination?	2—Yes, reliable determination of the resectability is possible using staging laparoscopy—34% 3—Yes, all surrounding structures should be assessed during staging laparoscopy—27% 4—Yes, all surrounding structures should be assessed only (!) in case of preoperative suspicion of infiltration/metastases—20%	3—Yes, all surrounding structures should be assessed during staging laparoscopy Reason: Staging laparoscopy is a crucial tool for determining resectability, especially in advanced gastric cancer. It allows for direct visualization of peritoneal surfaces, surrounding structures, and ascites, providing valuable information about peritoneal metastases or invasion of adjacent structures. Comprehensive assessment of all surrounding structures during the procedure increases the reliability of the resectability evaluation.
22	The (technical) resectability of the primary tumour by means of gastrectomy should be assessed during the staging laparoscopy by evaluation of the surrounding structures:	1—Yes, all surrounding structures should be assessed during staging laparoscopy—39% 2—Yes, all surrounding structures should be assessed only (!) in case of preoperative suspicion of infiltration/metastases on imaging—34%	1—Yes, all surrounding structures should be assessed during staging laparoscopy Reason: Staging laparoscopy is a crucial tool for assessing the technical resectability of the primary tumor. A thorough evaluation of all surrounding structures allows for a comprehensive determination of local invasion, peritoneal metastases, or other factors affecting resectability. This ensures that no potentially resectable tumor is overlooked and that unnecessary laparotomies for unresectable cases are avoided.
24	An expert opinion is to be formed on the nature of the operation. The operating surgeon should ascertain details about the extent of disease, including nodal involvement:	1—Curative—46% 4—Resection technically possible, but oncologically not justified due to substantial risk of R1/2 resection—26%	4—Resection technically possible, but oncologically not justified due to substantial risk of R1/2 resection Reason: This choice reflects a nuanced understanding of surgical decision-making. Even if resection is technically possible, the oncological outcome must be the priority. If achieving R0 resection (curative intent) is unlikely due to extensive disease or nodal involvement, surgery may not be justified, as R1/2 resection provides limited survival benefit and exposes the patient to unnecessary risks.

**Table 5:** Comparison of Surgeon Responses Before and After Exposure to AI Recommendations.

Question 1: Resectability is to be decided based on preoperative imaging, staging laparoscopy findings and intraoperative findings (clinical stages, cTNM, cStage). Do you think terms technical resectability & oncological resectability are different:(multiple choices are relevant)			
Answers	Before AI (first round) N = 59	After AI (second round) N = 48	p
Yes, they are	47 (79.66%)	38 (79.17%)	1.0000
No, they are not	4 (6.78%)	4 (8.33%)	1.0000
Curative gastrectomy (performed with the intention of cure) refers only to oncological resectability.	38 (64.41%)	23 (47.92%)	0.1163
Palliative gastrectomy (when cure is not possible) is performed to relieve patients (serious) symptoms only when it may be done safely	38 (64.41%)	28 (58.33%)	0.5536
Reduction gastrectomy should be performed for oligometastatic disease	1 (1.69%)	2 (4.17%)	0.5862
Reduction gastrectomy should be performed for oligometastatic disease with partial response to preoperative chemotherapy	14 (23.73%)	11 (22.92%)	1.0000
Other (please specify)	1 (1.69%)	0 (0%)	1.0000
Question 2: Determining whether the surgical margins are clear of cancer cells by examining the resected tissue (frozen section) helps assess the oncological effectiveness of the procedure. Inability to achieve clear resection margins is a criterion of non-resectability:			
Answers	Before AI (first round) N = 58	After AI (second round) N = 48	p
Yes, but only macroscopically positive margins (R2)	14 (24.14%)	19 (39.58%)	0.0967
Yes, but only when you prove it with an intraoperative frozen section	18 (31.03%)	12 (25.00%)	0.5235
No, even when intraoperative frozen section is positive, because final pathology of the resection specimen may be different (clear margins)	10 (17.24%)	6 (12.50%)	0.5912
No, even R2 resection is justified if it may eliminate symptoms	3 (5.17%)	3 (6.25%)	1.0000
Resections margins should be clear at every cost, and they are not a non-resectability criterion	10 (17.24%)	8 (16.67%)	1.0000
Other (please specify)	3 (5.17%)	0 (0%)	0.2496
Question 3: Achieving R0 resection status is critical for optimizing survival outcomes, despite the inherent risks and complications associated with extensive surgical interventions. Therefore, monitoring the occurrence of postoperative complications (morbidity) and mortality provides insight into the quality of perioperative care.			
Answers	Before AI (first round) N = 58	After AI (second round) N = 48	p
Risk of morbidity/mortality should always be in mind before decision of multivisceral resection	37 (63.79%)	30 (62.50%)	1.0000
Risk of morbidity/mortality is independent of multivisceral resection	1 (1.72%)	2 (4.17%)	0.5887
Risk of morbidity/mortality associated with multivisceral resection may be outweighed by the benefits of improved survival after curative gastrectomy	19 (32.76%)	16 (33.33%)	1.0000
Risk of morbidity/mortality associated with multivisceral resection is so high that it cannot outweighed by the benefits of improved survival after R0 resection	1 (1.72%)	0 (0.00%)	1.0000
Question 4: Tracking the rate of morbidity/mortality following gastrectomy is an important measure of surgical quality and patient safety. Whether these outcome measures should influence a decision on performance of gastrectomy with multivisceral resection?			
Answers	Before AI (first round) N = 58	After AI (second round) N = 48	p
Multivisceral resections for locally advanced gastric cancer should only be undertaken in centres that carefully monitor their morbidity/mortality associated with gastrectomy and multivisceral resection	14 (24.14%)	5 (10.42%)	0.0791
Multivisceral resections for locally advanced gastric cancer should only be undertaken in centres that demonstrate acceptably low morbidity/mortality associated with gastrectomy and multivisceral resection	6 (10.34%)	4 (8.33%)	1.0000
Multivisceral resections for locally advanced gastric cancer should only be undertaken in centres that carefully monitor their own results and can demonstrate acceptably low morbidity/mortality associated with gastrectomy and multivisceral resection	35 (60.34%)	37 (77.08%)	0.0941
Multivisceral resections for locally advanced gastric cancer should only be undertaken in large volume centres irrespectively of their own results	2 (3.45%)	2 (4.17%)	1.0000
Multivisceral resections are not justified in the surgical treatment of locally advanced gastric cancer	0 (0.00%)	0 (0.00%)	N/a
Other (please specify)	1 (1.72%)	0 (0.00%)	1.0000
Question 5: A shorter hospital stay is generally associated with better surgical outcomes and may indicate a more efficient recovery process. Therefore, duration of hospital stay for oncological gastrectomy should be below:			
Answers	Before AI (first round) N = 58	After AI (second round) N = 48	p
10 days	15 (25.86%)	16 (33.33%)	0.5203
11 days	1 (1.72%)	0 (0.00%)	1.0000
12 days	3 (5.17%)	0 (0.00%)	0.2496
14 days	3 (5.17%)	1 (2.08%)	0.6247
75th percentile by year	0 (0.00%)	3 (6.25%)	0.0897
Depends on total, distal or proximal gastrectomy	10 (17.24%)	19 (39.58%)	0.0155 *
Depends on splenectomy, pancreatectomy, any multivisceral resection, or HIPEC	20 (34.48%)	9 (18.75%)	0.0829
Other (please specify)	6 (10.34%)	0 (0%)	0.0309 *
Question 6: Is gastrectomy with multivisceral resection justified for cT4b tumours (that invades adjacent structures such as spleen, transverse colon, liver, diaphragm, pancreas, abdominal wall, adrenal gland, kidney, small intestine, or retroperitoneum):			
Answers	Before AI (first round) N = 58	After AI (second round) N = 48	p
Yes, it is always justified	0 (0.00%)	1 (2.08%)	0.4528
No, never	3 (5.17%)	0 (0.00%)	0.2496
Only if R0 is anticipated to achieve	22 (37.93%)	15 (31.25%)	0.5417
Only if the patient’s general condition allows for the success of the operation	4 (6.90%)	4 (8.33%)	1.0000
Only if surgical team can prove low morbidity/mortality, as well as the multidisciplinary team can ensure that all aspects of the patient’s condition are considered.	26 (44.83%)	28 (58.33%)	0.1783
Other (please specify)	3 (5.17%)	0 (0.00%)	0.2496
Answers	Before AI (first round) N = 58	After AI (second round) N = 48	p
Question 7: Is gastrectomy with metastasectomy justified for cM1 tumours:			
Yes, it is always justified	0 (0.00%)	0 (0.00%)	N/a
No, never	0 (0.00%)	2 (4.17%)	0.2027
Only if R0 or complete cytoreduction group (CCR 0–1) is anticipated to achieve	27 (46.55%)	25 (52.08%)	0.6966
Only if the stage does not exceed (generally accepted) definition of the oligometastatic disease	26 (44.83%)	21 (43.75%)	1.0000
Only if the resection can be done on one stage	0 (0.00%)	0 (0.00%)	N/a
Other (please specify)	5 (8.62%)	0 (0.00%)	0.0621
Answers	Before AI (first round) N = 58	After AI (second round) N = 48	p
Question 8: Is gastrectomy with peritonectomy justified for P1 tumours (with overt peritoneal metastasis):			
Yes, but only for P1a (previous P1—i.e., disseminating metastasis to the region directly adjacent to the peritoneum of the stomach, including the greater omentum; not distant peritoneum or ovarian metastases)	9 (15.52%)	4 (8.33%)	0.3746
Yes, but only for PCI below 7, irrespectively of histologic type	5 (8.62%)	2 (4.17%)	0.4525
Yes, but only for PCI below 12, with poorly cohesive type below 7	9 (15.52%)	7 (14.58%)	1.0000
Only if R0 is anticipated to achieve	2 (3.45%)	3 (6.25%)	0.6566
Only if complete cytoreduction (CC0/CC1) is to be achieved	5 (8.62%)	10 (20.83%)	0.0949
Only if the resection can be supplemented with HIPEC	1 (1.72%)	0 (0.00%)	1.0000
Only if the resection is preceded by major regression under the induction (neoadjuvant) systemic [with/without intraperitoneal therapy (i.p. normothermic paclitaxel/PIPAC)] therapy	21 (36.21%)	22 (45.83%)	0.3287
Other (please specify)	6 (10.34%)	0 (0.00%)	0.0309 *
Answers	Before AI (first round) N = 58	After AI (second round) N = 48	p
Question 9: Is gastrectomy with extensive peritoneal lavage justified for CY1 tumours (with positive peritoneal cytology):			
No, never	2 (3.45%)	5 (10.42%)	0.2404
No, extensive peritoneal lavage is justified only for CY0 and positive molecular cytology (CEA, OSNA etc.)	4 (6.90%)	2 (4.17%)	0.6872
Yes, but only if R0 is anticipated to achieve	8 (13.79%)	9 (18.75%)	0.5974
Only if the resection can be supplemented with HIPEC	10 (17.24%)	6 (12.50%)	0.5912
Only if the resection is preceded by conversion to negative peritoneal cytology under the induction (neoadjuvant) systemic [with/without intraperitoneal therapy (i.p. normothermic paclitaxel/PIPAC)] therapy	32 (55.17%)	26 (54.17%)	1.0000
Other (please specify)	2 (3.45%)	0 (0.00%)	0.4997
Answers	Before AI (first round) N = 58	After AI (second round) N = 48	p
Question 10: Is gastrectomy with extended (D2plus/D3) lymphadenectomy justified for N3 bulky lymph node metastasis:			
No, never	3 (5.17%)	3 (6.25%)	1.0000
Only if the resection is preceded by major regression under the induction (neoadjuvant) systemic (immuno-)therapy	36 (62.07%)	32 (66.67%)	0.6867
Yes, but only if R0 is anticipated to achieve	8 (13.79%)	7 (14.58%)	1.0000
Yes, but only if para-aortic lymph nodes (station #16) can be removed electively	1 (1.72%)	3 (6.25%)	0.3265
Yes, but only if enlarged para-aortic lymph nodes (station #16) can be effectively removed	5 (8.62%)	3 (6.25%)	0.7263
Only if the resection can be supplemented with HIPEC	1 (1.72%)	0 (0.00%)	1.0000
Other (please specify)	4 (6.90%)	0 (0.00%)	0.1246
Answers	Before AI (first round) N = 56	After AI (second round) N = 47	p
Question 11: Evaluating the extent and thoroughness of lymph node dissection is important for staging and determining the prognosis of the patient. If a case is borderline resectable at your initial (after exploration) evaluation, should it modify the extent of ultimate lymphadenectomy:			
No, it should be reduction gastrectomy (D0)	1 (1.79%)	0 (0.00%)	1.0000
No, it should be D1 (perigastric lymph nodes)	0 (0.00%)	0 (0.00%)	N/a
No, it should be D1 plus (depends on localisation of the primary tumour)	0 (0.00%)	0 (0.00%)	N/a
No, it should be always just D2	12 (21.43%)	12 (25.53%)	0.6470
Yes, it should be D2 plus (extraregional lymph nodes)	9 (16.07%)	4 (8.51%)	0.3730
Yes, it should be D2 plus (extraregional lymph nodes) if there is a good response to preoperative therapy	30 (53.57%)	31 (65.96%)	0.2313
Other (please specify)	4 (7.14%)	0 (0.00%)	0.1234
Answers	Before AI (first round) N = 55	After AI (second round) N = 42	p
Question 12: Extensive nodal swelling along the major branched arteries or para-aortic lymph node swelling is called bulky-N disease. If large arteries are surrounded by bulky nodal metastatic tissue, do you consider the case resectable? Please indicate non-resectability (multiple choices are relevant):			
Coeliac axis	26 (47.27%)	24 (57.14%)	0.4132
Common hepatic a.	17 (30.91%)	19 (45.24%)	0.2032
Left gastric a.	6 (10.91%)	3 (7.14%)	0.7275
Proximal portion of splenic a.	4 (7.27%)	3 (7.14%)	1.0000
any of the above	12 (21.82%)	10 (23.81%)	0.8124
Other (please specify)	17 (30.91%)	0 (0%)	<0.0001 *
Answers	Before AI (first round) N = 57	After AI (second round) N = 48	p
Question 13: Would you call it multivisceral resection? Gastrectomy with:(multiple choices are relevant)			
Omentectomy (both, major and minor omentum)	2 (3.51%)	1 (2.08%)	1.0000
Bursectomy	2 (3.51%)	2 (4.17%)	1.0000
Right and/or left diaphragmatic crus	14 (24.56%)	15 (31.25%)	0.5138
Splenectomy	45 (78.95%)	37 (77.08%)	0.8180
Distal pancreatectomy	52 (91.23%)	48 (100.00%)	0.0611
Liver resection with one/two liver segments	49 (85.96%)	45 (93.75%)	0.2212
Liver resection with three or more liver segments	36 (63.16%)	37 (77.08%)	0.1407
Pancreatoduodenectomy	49 (85.96%)	45 (93.75%)	0.2212
Colonic resection (hemicolectomy or transversectomy)	55 (96.49%)	48 (100.00%)	0.4989
Salpingo-oophorectomy with or without hysterectomy	41 (71.93%)	38 (79.17%)	0.4973
Other (please specify)	1 (1.75%)	0 (0.00%)	1.0000
Answers	Before AI (first round) N = 56	After AI (second round) N = 47	p
Question 14: Please indicate structures (that may be directly invaded; cT4b) to be resected if primary tumour is located at the esophago-gastric junction:(multiple choices are relevant)			
Intrathoracic esophagus (below carina)	32 (57.14%)	33 (70.21%)	0.2196
Lower esophagus (transhiatally)	41 (73.21%)	36 (76.60%)	0.8207
Whole stomach	26 (46.43%)	32 (68.09%)	0.0305 *
Distal pancreas	25 (44.64%)	26 (55.32%)	0.3254
Spleen	31 (55.36%)	30 (63.83%)	0.4253
Proximal one/third of the stomach	23 (41.07%)	26 (55.32%)	0.1696
Proximal two/thirds of the stomach	14 (25.00%)	20 (42.55%)	0.0917
Left liver lobe	31 (55.36%)	33 (70.21%)	0.1545
Other (please specify)	5 (8.93%)	0 (0.00%)	0.0612
Answers	Before AI (first round) N = 56	After AI (second round) N = 48	p
Question 15: Please indicate structures (that may be directly invaded; cT4b) to be resected if primary tumour is located at the proximal stomach (not invading esophago-gastric junction): (multiple choices are relevant)			
Lower esophagus (transhiatally)	33 (58.93%)	31 (64.58%)	0.6863
Whole stomach	38 (67.86%)	38 (79.17%)	0.2678
Distal pancreas	35 (62.50%)	37 (77.08%)	0.1372
Spleen	40 (71.43%)	36 (75.00%)	0.8250
Proximal one/third of the stomach	10 (17.86%)	16 (33.33%)	0.1108
Proximal two/thirds of the stomach	14 (25.00%)	19 (39.58%)	0.1403
Left liver lobe	33 (58.93%)	34 (70.83%)	0.2246
Other (please specify)	6 (10.71%)	0 (0.00%)	0.0295 *
Answers	Before AI (first round) N = 56	After AI (second round) N = 48	p
Question 16: Please indicate structures (that may be directly invaded; cT4b) to be resected if primary tumour is located at the middle third of the stomach:(multiple choices are relevant)			
Lower esophagus (transhiatally)	9 (16.07%)	18 (37.50%)	0.0150 *
Whole stomach	45 (80.36%)	41 (85.42%)	0.6063
Distal pancreas	41 (73.21%)	39 (81.25%)	0.3606
Spleen	43 (76.79%)	36 (75.00%)	1.0000
Proximal one/third of the stomach	10 (17.86%)	18 (37.50%)	0.0284 *
Left liver lobe	34 (60.71%)	36 (75.00%)	0.1451
Distal stomach (antrectomy)	10 (17.86%)	12 (25.00%)	0.4715
Other (please specify)	4 (7.14%)	0 (0.00%)	0.1222
Answers	Before AI (first round) N = 56	After AI (second round) N = 48	p
Question 17: Please indicate structures (that may be directly invaded; cT4b) to be resected if primary tumour is located at the distal stomach:(multiple choices are relevant)			
Whole stomach, if poorly cohesive carcinoma	36 (64.29%)	32 (66.67%)	0.8385
Distal pancreas	23 (41.07%)	26 (54.17%)	0.2375
Spleen	20 (35.71%)	24 (50.00%)	0.1663
Distal three/thirds of the stomach (small remnant)	33 (58.93%)	33 (68.75%)	0.3160
Distal stomach	24 (42.86%)	20 (41.67%)	1.0000
Antrum of the stomach	18 (32.14%)	17 (35.42%)	0.8356
Colon	41 (73.21%)	42 (87.50%)	0.0884
Head of a pancreas	30 (53.57%)	32 (66.67%)	0.2295
Other (please specify)	6 (10.71%)	0 (0.00%)	0.0295 *
Answers	Before AI (first round) N = 57	After AI (second round) N = 48	p
Question 18: Gastric adenocarcinomas are considered unresectable if there is evidence of locally advanced disease with:			
Disease infiltration of the root of the mesentery	46 (80.70%)	43 (89.58%)	0.2784
Para-aortic lymph nodes highly suspicious on imaging or confirmed by biopsy	14 (24.56%)	14 (29.17%)	0.6607
Invasion or encasement of major vascular structures (excluding the splenic vessels)	41 (71.93%)	35 (72.92%)	1.0000
N3 lymph node involvement	8 (14.04%)	9 (18.75%)	0.5983
Peritoneal involvement (including positive peritoneal cytology)	27 (47.37%)	26 (54.17%)	0.5585
Ovarian metastases	16 (28.07%)	14 (29.17%)	1.0000
Other (please specify)	4 (7.02%)	0 (0.00%)	0.1233
Answers	Before AI (first round) N = 56	After AI (second round) N = 48	p
Question 19: Gastric adenocarcinomas are considered unresectable if:			
the primary tumour shows extensive invasion to adjacent structures and cannot be separated from the surrounding normal tissues	30 (53.57%)	31 (64.58%)	0.3190
the primary tumour has encased major vascular structures	42 (75.00%)	37 (77.08%)	0.8226
the regional lymph nodes are fixed and fused into clusters	8 (14.29%)	12 (25.00%)	0.2142
presence of metastatic lymph nodes outside the scope of surgery	26 (46.43%)	36 (75.00%)	0.0048 *
presence of distant metastasis or intraperitoneal implantation (including positive peritoneal lavage fluid cytology)	23 (41.07%)	25 (52.08%)	0.3248
presence of distant metastasis or intraperitoneal implantation (excluding positive peritoneal lavage fluid cytology)	26 (46.43%)	26 (54.17%)	0.5554
Other (please specify)	4 (7.14%)	0 (0.00%)	0.1222
Answers	Before AI (first round) N = 56	After AI (second round) N = 48	p
Question 20: Would you consider all the above resectability criteria the same if a neoadjuvant systemic (chemo-/immuno) therapy had been used before?			
Yes, no matter preoperative treatment	18 (32.14%)	19 (39.58%)	0.5382
No, if preoperative immuno(chemo-)therapy was used	8 (14.29%)	9 (18.75%)	0.6011
Yes, but with cautious use of intraoperative pathology counselling	20 (35.71%)	8 (16.67%)	0.0450 *
More conservative approach is justified if clinical response had been achieved	5 (8.93%)	12 (25.00%)	0.0344 *
Just gastrectomy with adequate lymphadenectomy is required (for staging purposes)	4 (7.14%)	0 (0.00%)	0.1222
Other (please specify)	1 (1.79%)	0 (0.00%)	1.0000
Answers	Before AI (first round) N = 57	After AI (second round) N = 48	p
Question 21: Should staging laparoscopy be used for reliable resectability determination?			
No, reliable determination of the resectability is not possible using staging laparoscopy	8 (14.04%)	5 (10.42%)	0.7676
Yes, reliable determination of the resectability is possible using staging laparoscopy	19 (33.33%)	13 (27.08%)	0.5291
Yes, all surrounding structures should be assessed during staging laparoscopy	16 (28.07%)	12 (25.00%)	0.8258
Yes, all surrounding structures should be assessed only (!) in case of preoperative suspicion of infiltration/metastases for determining stage of the disease	11 (19.30%)	14 (29.17%)	0.2585
Reliable determination of neoplastic infiltration of surrounding structures can be decided only based on pathologic findings of the resected specimen	1 (1.75%)	4 (8.33%)	0.1760
Other (please specify)	2 (3.51%)	0 (0.00%)	0.4989
Answers	Before AI (first round) N = 57	After AI (second round) N = 47	p
Question 22: The (technical) resectability of the primary tumour by means of gastrectomy should be assessed during the staging laparoscopy by evaluation of the surrounding structures:			
Yes, all surrounding structures should be assessed during staging laparoscopy	23 (40.35%)	16 (34.04%)	0.5468
Yes, all surrounding structures should be assessed only (!) in case of preoperative suspicion of infiltration/metastases on imaging	19 (33.33%)	18 (38.30%)	0.6821
No, reliable determination of direct invasion of the surrounding structures is not possible during staging laparoscopy	10 (17.54%)	5 (10.64%)	0.4054
Anatomical structures for which otherwise no further dissection is necessary during standard gastrectomy (not multivisceral resection) should be inspected for direct invasion	5 (8.77%)	5 (10.64%)	0.7520
Reliable determination of neoplastic infiltration of surrounding structures can be decided only based on pathologic findings of the resected specimen	0 (0.00%)	3 (6.38%)	0.0890
Other (please specify)	0 (0.00%)	0 (0.00%)	N/a
Answers	Before AI (first round) N = 55	After AI (second round) N = 47	p
Question 23: Structures for which otherwise no further dissection is necessary during standard gastrectomy include:			
intrathoracic esophagus	22 (40.00%)	29 (61.70%)	0.0466 *
diaphragmatic crura	21 (38.18%)	24 (51.06%)	0.2319
liver	29 (52.73%)	35 (74.47%)	0.0260 *
spleen	28 (50.91%)	26 (55.32%)	0.6943
pancreas	28 (50.91%)	36 (76.60%)	0.0083 *
colon	27 (49.09%)	32 (68.09%)	0.0705
para-aortic lymph nodes	25 (45.45%)	33 (70.21%)	0.0161 *
ovaries	26 (47.27%)	35 (74.47%)	0.0081 *
greater omentum	16 (29.09%)	8 (17.02%)	0.1685
parietal peritoneum	24 (43.64%)	34 (72.34%)	0.0049 *
Other (please specify)	6 (10.91%)	0 (0.00%)	0.0295 *
Answers	Before AI (first round) N = 55	After AI (second round) N = 46	p
Question 24: An expert opinion is to be formed on the nature of the operation. The operating surgeon should ascertain details about the extent of disease, including nodal involvement:			
curative	25 (45.45%)	13 (28.26%)	0.0995
borderline curative	4 (7.27%)	5 (10.87%)	0.7282
palliative	0 (0.00%)	0 (0.00%)	N/a
resection technically possible, but oncologically not justified due to substantial risk of R1/2 resection	14 (25.45%)	21 (45.65%)	0.5466
resection technically not possible, even by means of extended multivisceral resection	5 (9.09%)	7 (15.22%)	0.3722
Other (please specify)	7 (12.73%)	0 (0.00%)	0.0149 *
Answers	Before AI (first round) N = 57	After AI (second round) N = 48	p
Question 25: At what point during the surgical process should the operating surgeon report whether the gastric cancer surgery is intended to be curative or palliative?			
At the beginning of the procedure	25 (43.86%)	21 (43.75%)	1.0000
After completion of the procedure (at the end)	7 (12.28%)	4 (8.33%)	0.7506
During the procedure, usually immediately following the resection	7 (12.28%)	9 (18.75%)	0.4200
Only after evaluation of the final pathology report of the resection specimen, irrespectively of the peritoneal (lavage) cytology findings	1 (1.75%)	3 (6.25%)	0.3296
Only after evaluation of the final pathology report of the resection specimen, including the peritoneal (lavage) cytology findings	14 (24.56%)	11 (22.92%)	1.0000
Other (please specify)	3 (5.26%)	0 (0.00%)	0.2484

* Statistical significance.

## Data Availability

No new data were created or analyzed in this study. Data sharing is not applicable to this article.

## Appendix A

**Table A1:** Summary of all questionnaire items from the ICRGC study.

Question No.	Question	Answer Options
1	Resectability is to be decided based on preoperative imaging, staging laparoscopy findings and intraoperative findings (clinical stages, cTNM, cStage). Do you think terms technical resectability & oncological resectability are different:(multiple choices are relevant)	Yes, they are No, they are not Curative gastrectomy refers only to oncological resectability Palliative gastrectomy is for symptom relief only when safe Reduction gastrectomy for oligometastatic disease Reduction gastrectomy after response to preoperative chemotherapy Other (please specify)
2	Determining whether the surgical margins are clear of cancer cells by examining the resected tissue (frozen section) helps assess the oncological effectiveness of the procedure. Inability to achieve clear resection margins is a criterion of non-resectability:	Yes, but only macroscopically positive margins (R2) Yes, but only when proven with intraoperative frozen section No, even when intraoperative frozen section is positive, because final pathology of the resection specimen may be different (clear margins) No, even R2 resection is justified if it may eliminate symptoms Resections margins should be clear at every cost, and they are not a non-resectability criterion Other (please specify)
3	Achieving R0 resection status is critical for optimizing survival outcomes, despite the inherent risks and complications associated with extensive surgical interventions. Therefore, monitoring the occurrence of postoperative complications (morbidity) and mortality provides insight into the quality of perioperative care.	Risk of morbidity/mortality should always be in mind before decision of multivisceral resection Risk of morbidity/mortality is independent of multivisceral resection Risk of morbidity/mortality associated with multivisceral resection may be outweighed by the benefits of improved survival after curative gastrectomy Risk of morbidity/mortality associated with multivisceral resection is so high that it cannot outweighed by the benefits of improved survival after R0 resection Other (please specify)
4	Tracking the rate of morbidity/mortality following gastrectomy is an important measure of surgical quality and patient safety. Whether these outcome measures should influence a decision on performance of gastrectomy with multivisceral resection?	Multivisceral resections for locally advanced gastric cancer should only be undertaken in centres that carefully monitor their morbidity/mortality associated with gastrectomy and multivisceral resection Multivisceral resections for locally advanced gastric cancer should only be undertaken in centres that demonstrate acceptably low morbidity/mortality associated with gastrectomy and multivisceral resection Multivisceral resections for locally advanced gastric cancer should only be undertaken in centres that carefully monitor their own results and can demonstrate acceptably low morbidity/mortality associated with gastrectomy and multivisceral resection Multivisceral resections for locally advanced gastric cancer should only be undertaken in large volume centres irrespectively of their own results Multivisceral resections are not justified in the surgical treatment of locally advanced gastric cancer Other (please specify)
5	A shorter hospital stay is generally associated with better surgical outcomes and may indicate a more efficient recovery process. Therefore, duration of hospital stay for oncological gastrectomy should be below:	10 days 11 days 12 days 14 days 75th percentile by year Depends on total, distal or proximal gastrectomy Depends on splenectomy, pancreatectomy, any multivisceral resection, or HIPEC Other (please specify)
6	Is gastrectomy with multivisceral resection justified for cT4b tumours (that invades adjacent structures such as spleen, transverse colon, liver, diaphragm, pancreas, abdominal wall, adrenal gland, kidney, small intestine, or retroperitoneum):	Yes, it is always justified No, never Only if R0 is anticipated to achieve Only if the patient’s general condition allows for the success of the operation Only if surgical team can prove low morbidity/mortality, as well as the multidisciplinary team can ensure that all aspects of the patient’s condition are considered. Other (please specify)
7	Is gastrectomy with metastasectomy justified for cM1 tumours?	Yes, it is always justified No, never Only if R0 or complete cytoreduction group (CCR 0–1) is anticipated to achieve Only if the stage does not exceed (generally accepted) definition of the oligometastatic disease Only if the resection can be done on one stage Other (please specify)
8	Is gastrectomy with peritonectomy justified for P1 tumours (with overt peritoneal metastasis):	Yes, but only for P1a (previous P1—i.e., disseminating metastasis to the region directly adjacent to the peritoneum of the stomach, including the greater omentum; not distant peritoneum or ovarian metastases) Yes, but only for PCI below 7, irrespectively of histologic type Yes, but only for PCI below 12, with poorly cohesive type below 7 Only if R0 is anticipated to achieve Only if complete cytoreduction (CC0/CC1) is to be achieved Only if the resection can be supplemented with HIPEC Only if the resection is preceded by major regression under the induction (neoadjuvant) systemic [with/without intraperitoneal therapy (i.p. normothermic paclitaxel/PIPAC)] therapy Other (please specify)
9	Is gastrectomy with extensive peritoneal lavage justified for CY1 tumours (with positive peritoneal cytology):	No, never No, extensive peritoneal lavage is justified only for CY0 and positive molecular cytology (CEA, OSNA etc.) Yes, but only if R0 is anticipated to achieve Only if the resection can be supplemented with HIPEC Only if the resection is preceded by conversion to negative peritoneal cytology under the induction (neoadjuvant) systemic [with/without intraperitoneal therapy (i.p. normothermic paclitaxel/PIPAC)] therapy Other (please specify)
10	Is gastrectomy with extended (D2plus/D3) lymphadenectomy justified for N3 bulky lymph node metastasis:	No, never Only if the resection is preceded by major regression under the induction (neoadjuvant) systemic (immuno-)therapy Yes, but only if R0 is anticipated to achieve Yes, but only if para-aortic lymph nodes (station #16) can be removed electively Yes, but only if enlarged para-aortic lymph nodes (station #16) can be effectively removed Only if the resection can be supplemented with HIPEC Other (please specify)
11	Evaluating the extent and thoroughness of lymph node dissection is important for staging and determining the prognosis of the patient. If a case is borderline resectable at your initial (after exploration) evaluation, should it modify the extent of ultimate lymphadenectomy:	No, it should be reduction gastrectomy (D0) No, it should be D1 (perigastric lymph nodes) No, it should be D1 plus (depends on localisation of the primary tumour) No, it should be always just D2 Yes, it should be D2 plus (extraregional lymph nodes) Yes, it should be D2 plus (extraregional lymph nodes) if there is a good response to preoperative therapy Other (please specify)
12	Extensive nodal swelling along the major branched arteries or para-aortic lymph node swelling is called bulky-N disease. If large arteries are surrounded by bulky nodal metastatic tissue, do you consider the case resectable? Please indicate non-resectability (multiple choices are relevant):	Coeliac axis Common hepatic a. Left gastric a. Proximal portion of splenic a. any of the above Other (please specify)
13	Would you call it multivisceral resection? Gastrectomy with: (multiple choices are relevant)	Omentectomy (both, major and minor omentum) Bursectomy Right and/or left diaphragmatic crus Splenectomy Distal pancreatectomy Liver resection with one/two liver segments Liver resection with three or more liver segments Pancreatoduodenectomy Colonic resection (hemicolectomy or transversectomy) Salpingo-oophorectomy with or without hysterectomy Other (please specify)
14	Please indicate structures (that may be directly invaded; cT4b) to be resected if primary tumour is located at the esophago-gastric junction: (multiple choices are relevant)	Intrathoracic esophagus (below carina) Lower esophagus (transhiatally) Whole stomach Distal pancreas Spleen Proximal one/third of the stomach Proximal two/thirds of the stomach Left liver lobe Other (please specify)
15	Please indicate structures (that may be directly invaded; cT4b) to be resected if primary tumour is located at the proximal stomach (not invading esophago-gastric junction): (multiple choices are relevant)	Lower esophagus (transhiatally) Whole stomach Distal pancreas Spleen Proximal one/third of the stomach Proximal two/thirds of the stomach Left liver lobe Other (please specify)
16	Please indicate structures (that may be directly invaded; cT4b) to be resected if primary tumour is located at the middle third of the stomach: (multiple choices are relevant)	Lower esophagus (transhiatally) Whole stomach Distal pancreas Spleen Proximal one/third of the stomach Left liver lobe Distal stomach (antrectomy) Other (please specify)
17	Please indicate structures (that may be directly invaded; cT4b) to be resected if primary tumour is located at the distal stomach: (multiple choices are relevant)	Whole stomach, if poorly cohesive carcinoma Distal pancreas Spleen Distal three/thirds of the stomach (small remnant) Distal stomach Antrum of the stomach Colon Head of a pancreas Other (please specify)
18	Gastric adenocarcinomas are considered unresectable if there is evidence of locally advanced disease with:	Disease infiltration of the root of the mesentery Para-aortic lymph nodes highly suspicious on imaging or confirmed by biopsy Invasion or encasement of major vascular structures (excluding the splenic vessels) N3 lymph node involvement Peritoneal involvement (including positive peritoneal cytology) Ovarian metastases Other (please specify)
19	Gastric adenocarcinomas are considered unresectable if:	the primary tumour shows extensive invasion to adjacent structures and cannot be separated from the surrounding normal tissues the primary tumour has encased major vascular structures the regional lymph nodes are fixed and fused into clusters presence of metastatic lymph nodes outside the scope of surgery presence of distant metastasis or intraperitoneal implantation (including positive peritoneal lavage fluid cytology) presence of distant metastasis or intraperitoneal implantation (excluding positive peritoneal lavage fluid cytology) Other (please specify)
20	Would you consider all the above resectability criteria the same if a neoadjuvant systemic (chemo-/immuno) therapy had been used before?	Yes, no matter preoperative treatment No, if preoperative immuno(chemo-)therapy was used Yes, but with cautious use of intraoperative pathology counselling More conservative approach is justified if clinical response had been achieved Just gastrectomy with adequate lymphadenectomy is required (for staging purposes) Other (please specify)
21	Should staging laparoscopy be used for reliable resectability determination?	No, reliable determination of the resectability is not possible using staging laparoscopy Yes, reliable determination of the resectability is possible using staging laparoscopy Yes, all surrounding structures should be assessed during staging laparoscopy Yes, all surrounding structures should be assessed only (!) in case of preoperative suspicion of infiltration/metastases for determining stage of the disease Reliable determination of neoplastic infiltration of surrounding structures can be decided only based on pathologic findings of the resected specimen Other (please specify)
22	The (technical) resectability of the primary tumour by means of gastrectomy should be assessed during the staging laparoscopy by evaluation of the surrounding structures:	Yes, all surrounding structures should be assessed during staging laparoscopy Yes, all surrounding structures should be assessed only (!) in case of preoperative suspicion of infiltration/metastases on imaging No, reliable determination of direct invasion of the surrounding structures is not possible during staging laparoscopy Anatomical structures for which otherwise no further dissection is necessary during standard gastrectomy (not multivisceral resection) should be inspected for direct invasion Reliable determination of neoplastic infiltration of surrounding structures can be decided only based on pathologic findings of the resected specimen Other (please specify)
23	Structures for which otherwise no further dissection is necessary during standard gastrectomy include:	intrathoracic esophagus diaphragmatic crura liver spleen pancreas colon para-aortic lymph nodes ovaries greater omentum parietal peritoneum Other (please specify)
24	An expert opinion is to be formed on the nature of the operation. The operating surgeon should ascertain details about the extent of disease, including nodal involvement:	curative borderline curative palliative resection technically possible, but oncologically not justified due to substantial risk of R1/2 resection resection technically not possible, even by means of extended multivisceral resection Other (please specify)
25	At what point during the surgical process should the operating surgeon report whether the gastric cancer surgery is intended to be curative or palliative?	At the beginning of the procedure After completion of the procedure (at the end) During the procedure, usually immediately following the resection Only after evaluation of the final pathology report of the resection specimen, irrespectively of the peritoneal (lavage) cytology findings Only after evaluation of the final pathology report of the resection specimen, including the peritoneal (lavage) cytology findings Other (please specify)
26	Please describe your experience with performing gastrectomy as an 1st operator:	Less than 5 years 5–10 years 10–15 years 15–20 years More than 20 years
27	What is your experience in laparoscopic gastrectomy:	No experience Less than 5 years 5–10 years 10–15 years 15–20 years More than 20 years
28	What is your experience in robotic gastrectomy:	No experience Less than 1 year 1–5 years 5–10 years 10–20 years More than 20 years
29	Do you perform staging laparoscopy in patients with gastric cancer?	Yes, routinely in patients with “non-early” GC who are not amenable to endoscopic treatment and who do not have clinically (imaging) detectable distant metastases (cM0) Yes, but only when non-resectability is suspected Yes, I start every gastrectomy for advanced GC with laparoscopy Yes, twice: as a staging procedure (before creation of treatment plan by the MDT), and after preoperative (neo-adjuvant) therapy—re-staging No, never
30	Do you perform extended D2plus/D3 lymphadenectomy with removal of extra-regional lymph nodes (i.e., para-aortic etc.)? (multiple choice)	Yes Sometimes If a positive para-aortic node is found (longest axis at least 10mm) at preoperative radiological examination In patients who have radiological evidence of positive para-aortic nodes before preoperative chemotherapy and then show clinical response at restaging examinations Only when lymph node metastases are proved (intraoperative pathology counselling) Even if these lymph nodes are not suspected for metastases Even if these lymph nodes are not suspected for metastases, but the patient shows clinical response to preoperative chemotherapy No, never
31	Do you perform multivisceral resection for cT4B gastric cancer?	Yes Sometimes Only when direct invasion of adjacent organ is proved (intraoperative pathology counselling) Even when direct invasion of adjacent organ is not suspected (preoperative imaging), but I have such a suspicion during gastrectomy No, never. It is not justified
32	Do you extend gastrectomy with metastasectomy (liver, adrenalectomy, ovaries, other organs) for M1 gastric cancer?	Yes Sometimes Only when M1 is proved by intraoperative pathology Only if it fulfils criteria of oligometastatic disease (OMEC definition) No, never. It is not justified
33	Do you extend gastrectomy with peritonectomy for P1 gastric cancer (with overt peritoneal metastasis)?	Yes, but only within a clinical trial Only when PCI is no more than 6 Only when PCI is no more than 12 for non-poorly cohesive tumours Only if the procedure can be supplemented with the HIPEC No, never. It is not justified
34	Do you perform gastrectomy for positive cytology (CY1) gastric cancer without peritoneal metastases at preoperative staging?	Yes always, but only within a clinical trial Only when positive cytology is reversed to negative by preoperative (induction) systemic therapy Even when peritoneal cytology still positive after preoperative systemic therapy Only if the procedure can be supplemented with the HIPEC No, never. It is not justified
35	What is your institution yearly gastrectomy volume?	<20 20–30 31–50 51—100 >100
36	What is your personal yearly gastrectomy volume?	<10 10–20 21–30 31—50 >50
37	What continent do you come from?	Asia North America South America Europe other
